# Traumatic Pneumocephalus Without Skull Fracture From a High-Voltage Electrical Injury

**DOI:** 10.7759/cureus.16700

**Published:** 2021-07-28

**Authors:** Katie L Priestley, Rachel E Bridwell, John C Beach, Erica M Simon, Garrett W Britton

**Affiliations:** 1 Emergency Medicine, Brooke Army Medical Center, Fort Sam Houston, USA; 2 School of Medicine, Uniformed Services University of the Health Sciences, Bethesda, USA; 3 Critical Care, United States Army Institute of Surgical Research, Fort Sam Houston, USA

**Keywords:** pneumocephalus, high-voltage, electrical injury, burn, trauma

## Abstract

Pneumocephalus, the presence of intracranial air, most commonly occurs secondary to a traumatic injury. Patients with simple pneumocephalus often present with nonspecific symptoms or with headaches. These patients may have little to no clinically relevant physical examination findings and can be managed conservatively. Tension pneumocephalus can present more acutely as a neurosurgical emergency. On physical examination, patients can present with neurologic deficits or papilledema. Computed tomography is the imaging modality of choice to detect intracranial air. We present a novel case of a simple pneumocephalus in the setting of a high-voltage electrical injury without evidence of displaced skull fracture or dural violation. The identification of unanticipated air within the cranial vault should prompt emergency physicians to determine its etiology which can guide treatment and disposition.

## Introduction

Pneumocephalus is defined as the presence of air within the cranial vault, including the epidural, subdural, or subarachnoid space; the brain parenchyma itself; or the ventricular system [1]. Although pneumocephalus can arise from congenital defects, malignancy, infection, or iatrogenic etiologies, it usually occurs secondary to trauma (e.g., basilar skull fractures, air sinus fractures, or dural-penetrating trauma) [1,2]. In a review of 295 cases of pneumocephalus, 74% were caused by trauma [3]. Pneumocephalus can be classified as either simple or tension. Patients with simple pneumocephalus often present asymptomatically or with mild complaints such as headache, whereas those with tension pneumocephalus may present more acutely [1]. Tension pneumocephalus develops when air accumulates via a ball-valve mechanism, allowing air to enter the fixed intracranial space without a means for it to exit [4,5]. We present a unique case of simple pneumocephalus following a high-voltage electrical injury without skull fracture or dural-penetrating trauma.

## Case presentation

**Figure 1:**
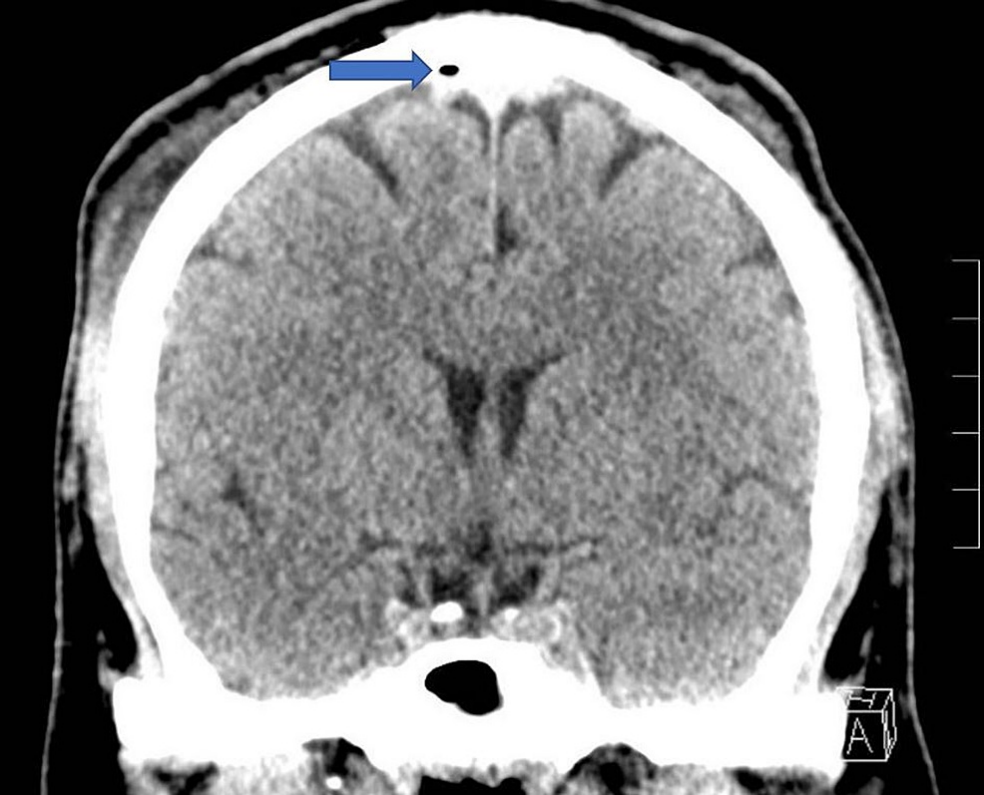
Coronal slice of a noncontrast CT demonstrating a small focus of air within the sagittal sinus without skull fracture as well as a slight displacement of the superior sagittal sinus from the adjacent skull, concerning for a possible small epidural hematoma measuring 4 mm in thickness without mass effect. CT: computed tomography

**Figure 2:**
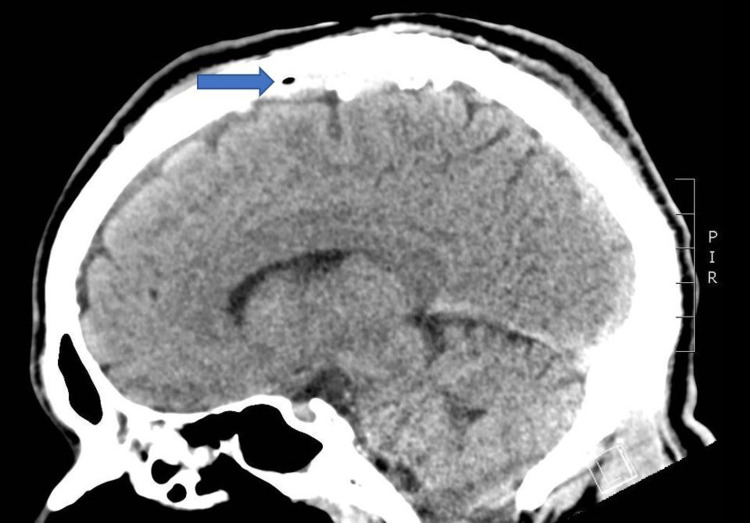
Sagittal slice of a noncontrast CT demonstrating a small focus of air within the sagittal sinus without skull fracture as well as a slight displacement of the superior sagittal sinus from the adjacent skull, concerning for a possible small epidural hematoma measuring 4 mm in thickness without mass effect. CT: computed tomography

A 34-year-old male electrician presented to the Emergency Department following a high-voltage electrical injury. He reportedly struck his head on a power line carrying approximately 1,000 volts of alternating current. His initial vital signs revealed a blood pressure of 135/40 mmHg, heart rate of 95 beats per minute, respiratory rate of 30 breaths per minute, oxygen saturation of 92% on 4 L/min by nasal cannula, and a temperature of 97.2°F. His Glasgow Coma Scale was 15. His secondary examination was notable for partial and full-thickness burns to his scalp, right forearm, and right lateral thigh, totaling 5% of the body surface area. Computed tomography (CT) of the head revealed a cephalohematoma, small epidural hematoma, and pneumocephalus without skull fracture (Figures 1, 2). Laboratory studies demonstrated an elevated creatinine kinase (CK) of 1,580 U/L. Urinalysis showed 2+ hemoglobin and two red blood cells per high-power field, consistent with rhabdomyolysis. His electrocardiogram showed sinus tachycardia, and a chest X-ray was concerning for pulmonary contusions. He was admitted to the Burn Intensive Care Unit and placed on a high-flow nasal cannula at 15 L/min and 100% FiO_2_ to treat his pneumocephalus. Repeat head CT on hospital day six did not show a significant change in pneumocephalus. Neurosurgery deferred surgical intervention given the absence of symptoms and neurologic deficits. During his hospital course, he developed compartment syndrome and underwent a right forearm fasciotomy. His CK normalized with fluid administration, and the plastic surgery team performed excision and grafting to his scalp and right forearm. He was discharged home on hospital day 21.

## Discussion

Patients with a simple pneumocephalus often present with a headache or nonspecific complaints [1-3,6]. A pathognomonic sign reported in a small percentage of cases is the “bruit hydro-aerique,” a splashing sound patients may report hearing during postural changes [4,6]. Patients with a simple pneumocephalus may have no relevant findings on physical examination. Patients with a tension pneumocephalus can have sensory deficits, papilledema, respiratory irregularities, or cardiac arrest if mass effect or herniation occurs [1]. CT of the head is the ideal imaging modality for identifying intracranial air, detecting as little as 0.55 mL of air [7].

The initial treatment of a simple pneumocephalus is conservative. It consists of bed rest with the head of the bed elevated at 30 degrees, analgesics, avoiding Valsalva maneuvers, and supplemental oxygen (5 L/min, 100% FiO_2_ is recommended) [1,6]. Supplemental oxygen increases the partial pressure of oxygen in the blood, replacing all other gases in the pneumocephalus with oxygen which improves resorption [1]. With conservative therapies, resolution occurs in approximately 85% of cases within one week [8]. Hyperbaric oxygen therapy has also been suggested, although data are limited [9]. Indications for neurosurgical intervention include symptomatic pneumocephalus, recurrent pneumocephalus, and persistent traumatic pneumocephalus lasting greater than one week [1]. Tension pneumocephalus is a neurosurgical emergency requiring immediate intervention (e.g., burr holes, needle aspiration, or surgical closure of the dural defect) [10]. Complications of untreated pneumocephalus include meningitis, seizures, brain abscesses, and herniation [1,10].

Pneumocephalus in the setting of high-voltage electrical injury has not been previously reported. In a review of the literature, pneumocephalus following a lightning strike occurred in a patient with a tympanic membrane rupture who was found to have a congenital defect in the petrous portion of their temporal bone [11]. In this case, the focus of intracranial air suggests that high-voltage electrical current may be an alternate etiology for pneumocephalus.

## Conclusions

Pneumocephalus is the presence of air in the cranial vault. There are a few sensitive physical findings unless the patient has the physiology for a tension pneumocephalus. CT imaging is the gold standard for identifying intracranial air. While pneumocephalus most often occurs secondary to trauma, this novel case demonstrates pneumocephalus without calvarial fracture or dural violation. Electrical current may be an alternate etiology for pneumocephalus. Given the serious complications of untreated pneumocephalus, emergency physicians should liberally consider head CTs in patients with high-voltage electrical injuries.
